# Delirium and Neuropsychological Recovery among Emergency General Surgery Survivors (DANE): study protocol for a randomized controlled trial and collaborative care intervention

**DOI:** 10.21203/rs.3.rs-3185716/v1

**Published:** 2023-09-07

**Authors:** Sanjay Mohanty, Emma Holler, Damaris Ortiz, Ashley Meagher, Anthony Perkins, Peggy Bylund, Babar Khan, Frederick Unverzagt, Hupuing Xu, Angela Ingraham, Malaz Boustani, Ben Zarzaur

**Affiliations:** Indiana University School of Medicine; Indiana University School of Medicine; Indiana University School of Medicine; Indiana University School of Medicine; Indiana University School of Medicine; Regenstrief Institute Inc; Indiana University School of Medicine; Indiana University School of Medicine; Indiana University School of Medicine; University of Wisconsin-Madison School of Medicine and Public Health; Indiana University School of Medicine; University of Wisconsin-Madison School of Medicine and Public Health

**Keywords:** postoperative delirium, emergency general surgery

## Abstract

**Background:**

Delirium is a complex neuropsychiatric syndrome which consists of acute and varying changes in cognition and consciousness. Patients who develop delirium are at increased risk for a constellation of physical, cognitive, and psychological disability long after the delirium has ended. Collaborative care models integrating primary and specialty care in order to address patients with complex biopsychosocial needs has been demonstrated to improve outcomes in patients with chronic diseases. The purpose of this study is to evaluate the ability of a collaborative care model on the neuropsychologic recovery of delirium survivors following emergency surgery.

**Methods:**

This protocol describes a multicenter (eight hospitals in three states) randomized controlled trial in which 528 patients who develop delirium following emergency surgery will be randomized to either a collaborative care model or usual care. The efficacy of the collaborative care model on cognitive, physical, and psychological recovery in this delirium survivors will then be evaluated over eighteen months.

**Discussion:**

This will be among the first randomized clinical trials in postoperative delirium survivors evaluating an intervention designed to mitigate the downstream effects of delirium and improve the neuropsychologic recovery after surgery. We hope that the results of this study will add to and inform strategies to improve postoperative recovery in this patient group.

**Trial registration:**

NCT05373017 (clinicaltrials.gov)

## Background

Delirium is a neuropsychiatric syndrome which is characterized by acute and fluctuating changes in cognition and consciousness.^1^ It is a devastating, common complication which affects more than one-quarter of patients following emergency surgery. With over 1.5 million emergent and urgent surgeries occurring each year along with an increasing population of older people, delirium and the subsequent neurocognitive decline following surgery is a serious public health problem.

Postoperative delirium is also associated with a constellation of adverse downstream effects, which includes increased healthcare resource utilization, lower quality of life, loss of functional independence, and increased risk of Alzheimer disease and related dementias (ADRD).^1,2^ These patients often have complex, cognitive, physical, and psychological morbidity and increased care burden, which may include visits to several healthcare providers and allied health professionals (e.g., physical therapy, speech therapy) and in home care services. These patients are at particular risk of worse outcomes during care transitions due to fragmentation of care. Care fragmentation in this group is associated with a high risk of readmission, falls, drug overprescribing, poor quality of life, and further decline in function.

The National Academy of Science, Engineering and Medicine (NASEM) emphasizes that care after acute care hospitalization should be evidence based, patient-centered, continuous, and without fragmentation. The academy suggests that collaborative care models are a key way to achieve a rapid and full recovery in patient populations that are vulnerable to breakdowns in coordination of post-acute care.^3^ Several randomized controlled trials demonstrate that collaborative care models enhanced quality of life, reduced care fragmentation, and improved psychological morbidity among complex patient populations.^4–9^ However, there is no effective, personalized, and scalable care model to manage the morbidity of emergency or unplanned postoperative delirium survivors. Building on our experience designing collaborative care interventions for vulnerable patient populations, we designed a novel collaborative care model that leverages telehealth to target older delirium survivors after emergency surgery. The proposed randomized controlled trial will evaluate the feasibility and superiority of this collaborative care model on the cognitive, physical, and psychological recovery of delirium survivors following emergency surgery when compared to usual care.

## Methods and design

### Study population

This is a multicenter, prospective randomized controlled trial which will evaluate the efficacy of a collaborative care model, delivered via telehealth, in improving the cognitive, functional, and psychological recovery of older emergency general surgery delirium survivors. Our study sites span three states in the Midwest and includes a safety net hospital serving many underprivileged and minority patients (Eskenazi Hospital), four community hospitals (Indiana University Health (IUH) North, IUH West, Meriter Hospital, and Swedish American Hospital) and three tertiary academic health centers (IUH Methodist, IUH University Hospital, and the University of Wisconsin Health University Hospital). Together, our team has assessed close to 8,000 intensive care unit patients as well as older injured patients for eligibility and enrolled nearly 1000 patients into two NIH-funded trials. For this study, we plan to randomize 528 patients.

### Inclusion and exclusion criteria

Patients will be eligible for inclusion in this study if they are English-speaking, aged 65 years and older, admitted to one of our participating hospital, and have undergone one of the following procedures in an emergent, urgent, or unplanned fashion: colectomy, small bowel resection, repair of peptic ulcer disease, lysis of adhesions, laparotomy, esophagectomy, liver resection, hepatopancreatobiliary surgery, gastrectomy, hernia repair, cystectomy, gynecological surgery, major vascular procedures, and major orthopedic (e.g., fractures) procedures. They will also need to be able to provide consent or have a legally authorized representative to provide consent, have access to a telephone or internet connected computer or smart device, be discharged to home or sub-acute rehabilitation, and have had at least one episode of delirium in the period prior to discharge from acute care.

Broadly speaking, exclusion criteria will target patients in overall declining health, for whom it may be unethical to intervene, or for whom there is no expectation of full recovery. These include: a self-reported diagnosis of cancer with short life expectancy (< 6 months); current chemotherapy or radiation therapy confirmed in the electronic medical record (EMR); a history of Alzheimer’s disease and related dementias (ADRD) and other neurodegenerative disorders such as Parkinson’s disease or vascular dementia; incarcerated or unhoused status at the time of enrollment; acquired neurologic injury (stroke, traumatic brain injury, cerebral edema/swelling, anoxic brain injury, or any other acute/subacute neurologic deficit) as the admitting diagnosis or during the course of the hospital stay; a history of bipolar disorder or schizophrenia confirmed by EMR; current alcohol consumption >5 drinks per day by self-report and/or confirmed by EMR; and history of drug abuse within the last three months.

### Ethics and informed consent

Both the University of Wisconsin and Indiana University Institutional Review Boards (IRB) approved this randomized controlled trial. This trial is registered on ClinicalTrals.gov (NCT05373017). Our team will approach all eligible participants prior to discharge for enrollment. We will obtain informed consent from the patient or a legally authorized representative by a trained Research Assistant (RA) or Research Coordinator. After explanation of the study, all potential trial participants will be given a printed and/or electronic consent document and HIPAA release form. All personnel will be IRB-approved and trained on confidentiality, ethical research practices, and enrollment procedures. All participant data will be collected, shared, and maintained within IRB and HIPAA guidelines, and only approved study team members will have access to collected data. The data will be stored in a secure Research Electronic Data Capture (REDCap) database on password-protected University of Wisconsin server.

### Adverse events

Adverse events will reported to the co-Principal Investigators, statisticians, and the Data Safety Monitoring Board (DSMB) as well as the IRB, with appropriate action as deemed per policy. We will also formally document such occurrences. Re-hospitalizations, emergency department visits, and deaths will be reported via EMR notifications or from family members/caregivers at the time of outcome assessments, which are provided to the DSMB as regular meetings. Any other adverse events will be provided by reports from patients or their families / caregivers.

### Study design

This study is a multi-site, randomized controlled clinical trial. We will perform a two-arm, single-blinded study to evaluate the efficacy of the proposed DANE collaborative care model. At or near the time of discharge, enrolled patients will be randomized to receive the DANE intervention using a computer-generated randomization scheme, stratified by recruitment site and whether the procedure was primarily vascular, due to the known delirium risk with that operation type. The comparator will be standard care, which will consist of postoperative care visits and calls as would be typical at the respective facility and under the guidance of the care team. Patients will then be followed for 18 months (Fig. 2).

### Description of intervention

The DANE Recovery Model, our intervention, will occur across five phases corresponding to the documented stages of recovery after surgery.^10,11^ During the acute stage (0–1 month after surgery), patients will undergo the initial case review and two initial virtual visits, and we will develop the recovery care plan. During the recovery stage (2–6 months after surgery) and maintenance stage (7–12 months after surgery), the patients will undergo the interaction phase of the DANE Recovery Model. Based on our prior work in a critical care recovery program targeting ICU survivors, we know that improvements in recovery begin to manifest at six months, and we expect survivors of postoperative delirium to follow a similar trajectory.

At the initial case review, randomized patients, the care coordinator (CC) will introduce the DANE Recovery Model and determine the best mode of contact for future meetings. The time for the first visit will also be arranged, which marks the beginning of the recovery model.

After completing a pre-visit review, the CC will conduct the first virtual visit using a mobile visit technology to perform an initial assessment. The CC will also perform a social and community needs assessment, reconcile all prescribed and over-the-counter medications, and note all scheduled and recommended appointments with specialists, physical and occupational therapists, and other providers. The Healthy Aging Brain Care (HABC) Monitor will be used to evaluate and track cognitive, functional, and psychological symptoms of patient and caregiver stress and to trigger use of specific treatment protocols.^12^

The second virtual visit will include a detailed discussion of the individualized recovery care plan with the patient and caregivers, which will include the process of monitoring recovery progress, implementation of recovery protocols, distribution and explanation of educational materials, and connection to in-home and community resources as needed.

The twelve-month interaction period with patients and/or informal caregivers will include virtual visits, phone calls, email, and mail. The minimum amount of contact during this period will be every two weeks. At the end of the interaction period, the CC will transition the care to the patient’s primary care physician. Activities during these interactions will include: addressing any questions, collecting feedback, reviewing/reconciling medications and adherence, scheduled appointment review, HABC Monitor assessments, sleep monitoring, and facilitating access to any other resources in the community if needed.

Participants are free to withdraw from participation in the study at any time upon request. Those that do so will be informed that no new procedures will be performed after notice of study withdrawal. Information obtained up to that point will remain part of the study. The reason for withdrawal will also be recorded.

### Usual care

Patients randomized to usual care will also receive an initial review by the care coordinator prior at or near hospital discharge to confirm eligibility criteria, contact information, review discharge and rehabilitation plans, identify the primary care physician, and compose and send care transition information. Patients in the usual care group will receive no further interventions after this initial review.

### Randomization and blinding

On or within 48 hours of anticipated hospital discharge and after obtaining informed consent, the RA will obtain baseline measurements of physical function, depression, anxiety, quality of life, and cognitive ability. Baseline assessments Subjects will then be randomized in a 1:1 manner via a computer-generated randomization scheme, stratified by recruitment site and surgery type (vascular or non-vascular surgery). All subjects will receive the interventions described under usual care. Research staff performing assessments will be blinded to the intervention. Physicians and other providers caring for the patient will be made aware of the study but not its objectives. Intervention allocation will be coordinated centrally through the University of Wisconsin to minimize staff exposure to randomization assignment. Study staff will adhere to a strict study script during survey administration with patients and caregivers.

### Assessments and outcomes

The primary aims of this study are to evaluate the efficacy of the DANE recovery model to improve the cognitive recovery of older emergency surgery delirium survivors, as determined by the Repeatable Assessment of Neuropsychological Status (RBANS). Secondary aims include evaluation of physical and psychological recovery, as measured by the Short Physical Performance Battery, Medical Outcomes Study Short Form (SF-36), Patient Health Questionnare-9 (PHQ-9), and Generalized Anxiety Disorder Scale (GAD-7).

### Data collection

Chart review will be used to abstract relevant covariables. Baseline assessments will occur at or as close (within four weeks) to hospital discharge as possible. After baseline assessments, outcomes will be collected at 6-, 12-, and 18 months. We will make every effort to retain patients during the 18-month study period and complete all follow up instruments. We will attempt to do this in person. We will abstract data in a standardized fashion into a REDCap database. We will ensure data quality by independent abstraction and double data entry of 10% by the site PI or suitable designee. Any changes to the study protocol, including those related to inclusion and exclusion criteria, will be communicated to key personnel, trial participants, program officers, and IRBs.

### Description of study instruments

RBANS (Repeatable Battery for the Assessment of Neuropsychological Status)^13^: This is a commonly used battery of cognitive function. Originally developed as a screening instrument for older adults with suspected dementia, it is now used in a wider age range and variety of neuropsychological problems. This instrument is comprised of twelve subtests that aggregate to a Total and Index score across five domains: immediate memory, visuospatial-constructional, language, attention, and delayed memory. Index scores are then converted to age-based standard scores (M = 100, SD = 15). The test is versatile with four equivalent forms to allow for serial assessment. It takes approximately 30 minutes to administer.

#### SF-36^14,15^:

This widely validated and used study instrument evaluates eight components of quality of life: physical functioning, role-physical, bodily pain, general health, vitality, social functioning, role-emotional, and mental health. These components are aggregated into a physical component summary (PCS) and mental component summary (MCS). Differences of more than two points are considered clinically significant for both component summary scores.

#### PHQ-9^16^ and GAD-7^17^:

The former is a depression scale that consists of nine items with a total score of 0–27 and the latter is an anxiety scale that consists of seven items. These instruments are also widely validated with good internal consistency and reliability for the diagnosis of major depression and generalized anxiety disorder. A change of two points is considered clinically significant.

### Statistical analysis

We will compare randomization results to the planned randomization schedule. Baseline characteristics between the intervention and standard care arm will be compared using analysis of covariance (ANCOVA) for continuous variables and the Cochran-Mantel-Hansel statistic for categorical variables while adjusting for stratifying variables. Patients will be stratified by hospital and surgery (vascular v. non-vascular procedures). We will also examine the distributions of continuous variables in order to verify the normal distribution assumption, and use alternative procedures (transformation or non-parametric methods) if this assumption is violated. Frequency distributions of all categorical variables will also be examined, and exact inference procedures will be used in cases of zero or small cell size. Intention to treat analysis will be conducted for the primary and secondary aims.

For our primary outcome of cognitive recovery, will use mixed effects models with repeated RBANS scores collected at baseline, 6-, 12-, and 18-months as outcome measures. Group, time, a group by time interaction, and other baseline covariates found to be significantly different in univariate comparisons will be used as independent variables. We will adjust for stratification variables. We will also conduct post-hoc comparisons at each follow-up time following a significant interaction between group and time to determine the time when a group difference can be detected. Parameter estimation and inference for the mixed-effects models are conducted using the maximum likelihood approach which are robust under many missing data mechanisms.

For our secondary aims of physical and psychological recovery, we will develop separate mixed effects models for physical function scores (SPPB and physical component score on the SF-36) collected at baseline, 6-, 12-, and 18-months as the outcome measures for the former; and PHQ-9, GAD-7, or mental component score on the SF-36 for the latter. In both sets of models, we will use same independent variable selection and stratification variables as in our primary outcome.

In an exploratory analysis, we will plan to we will investigate heterogeneity of treatment effects whenever a treatment effect is detected in order to determine if the treatment has different effects in various subgroups defined by sex, race/ethnicity, socioeconommc status, and age.

We completed a sample size calculation using a mixed effects model and adjusted for correlations among repeated measures. Assuming a base correlation of 0.3 and a decay rate of 0.1 in a linear exponent autoregressive correlation structure for repeated measures and effect sizes of 0.2SD at 6-months, 0.4SD at 12-months and a maintained effect size of 0.4SD at 18-months, 185 participant per group is required to yield a power of 80.6% to detect differences in change in RBANS between the intervention and usual care groups with type I error rate at α= 0.05. Assuming a 30% attrition rate, we need to enroll 264 patients per group into the study with the total enrollment target size of 528. Similar power will be achieved for the secondary outcome measures. Power estimates were calculated using the GLMPower procedure in SAS 9.4.

### Data safety monitoring board and data safety

This trial will be monitored every six months by a Data Safety Monitoring Board (DSMB) and reviewed annually by the University of Wisconsin IRB. The board will consist of three members: a surgeon, a biostatistician, and a safety officer. This team will be separate from the study team without competing interests. Review reports will include any necessary additional actions as necessary (e.g., corrective intervention, ad hoc review, stopping rule, communication requirements with study participants or investigators). The DSMB will meet twice annually either in-person or by teleconference call to review study progress, data quality, and participants’ safety. We will submit quarterly reports to the DSMB in addition to these in person meetings. Our investigator group will continue to meet on a weekly basis to discuss trial progress, weekly enrollments, specific concerns, and amendments to trial protocol. All clinical outcomes will by systematically tracked throughout the study period. Clinical outcomes including death, hospital or ICU readmission, depressive symptoms, anxiety symptoms, agitation and behavioral disturbances, and fall/mobility problems will be tracked as clinical outcomes and are not required to be reported as adverse events. Adverse events will be classified in terms of severity, expectedness, and relatedness. The study coordinator and biostatistician will generate reports for the PI, safety officer, and DSMB which will contain summaries of adverse events, complaints, retention, intervention compliance, and protocol violations, including how they were handled. Adverse events will be reported within 24 hours to the study team, to the IRB within 14 business days, and the NIA within 48 hours. Summary of all reportable adverse events and clinical outcomes will be reported at least twice a year (initially quarterly and then every six months beginning at a time determine by the DSMB).

All electronic data is password and firewall security protected, and any data set containing patient identifiers will be managed by the investigators, sub-investigators, or a designated programmer. Data sets with patient identifiers will be stored indefinitely and for a minimum of seven years on limited-access folders on UW and IU servers in which only key study personnel listed on the IRB application will have access.

### Special considerations

#### Missing Data

We anticipate two types of missing data in this trial. The first are those that are due to mortality during the follow up period, and the second is due to loss to follow up. Our previous work in ICU survivors and other work in the surgical literature has shown that most post-ICU deaths happen within the first 30 days of discharge. We do not expect death rates to be different between randomization groups, and we will monitor death closely during the trial using both follow up contact and information from the EMR. The second type of missing data comes from participant withdrawal during follow-up or inability to contact. This may be more frequent in the usual care group than in the intervention group due to infrequent contact when compared to the intervention group. The mixed effects approach we propose is robust under the missing at random assumption (i.e. the probability of missing is unrelated to the missing outcomes). However, we will compare baseline characteristics of patients with missing outcomes to those with complete outcome ascertainment to detect violation of this assumption. We will also perform sensitivity analyses using various methods of imputation or a full parametric likelihood approach assuming various patterns of missing data.

#### Recruitment and retention

Patients who meet screening criteria will be approached while in the hospital following their operation. They will be screened for development of delirium with EMR documentation or with the CAM-ICU. Surrogate consent will be obtained for patients deemed unable to provide their own consent. Based on our experience with previous studies by our team, we assume a very conservative consent rate of 40% over the 36-month recruitment period. Thus, we expect to enroll at least 24 patients per month which is more than enough to meet our target enrollment of 15 patients per month in order to reach our target sample size of 528 subjects. To enhance consent and recruitment rates, we have established a strong relationship with the emergency general surgery clinical teams at all the enrolling hospitals and have enrolled subjects within these hospitals for previous studies. The primary investigator on this trial and the co-investigators who all have direct involvement in the care of emergency surgery patients.

With respect to retention, the usual care group presents the greatest challenge. As part of our quality control procedures during enrollment, we will monitor subjects’ perceptions about the risks and benefits of participation. We have and will continue to recruit research personnel who are representative of our target population and seek to identify individuals who have life experiences in the community from which they are recruiting. If retention drops below 80%, we will have staff follow up with the participants to troubleshoot issues and provide coaching if necessary. We will also institute gift card incentives and we will use fair subject payments contingent on the completion of the baseline, 6-, 12-, and 18-month follow up assessments. Finally, we have developed alternatives to face-to-face visits for measuring the SPPB and RBANS in the event that these visits are not possible.

#### Research Dissemination

Trial results will be prepared and submitted in manuscript form for peer-review. Trial results will also be communicated to participants, participating institutions, and presented at local and national surgical and aging conferences. Access to collected data will depend on the outcome to be measured.

Interested individuals will make a data request and prepare a project proposal that includes project goals, outcomes data to be collected, and the planned analysis. This project, if approved, will be carried out under the direction of the principal investigators after it has been deemed to fall under the intended use of this application. Publication authorship will be based on the relative scientific contributions of the PIs and key personnel.

## Discussion

This will be among the first trials testing the efficacy of a novel collaborative care intervention targeting survivors of postoperative delirium. The recovery of these patients is altered from those without delirium, and some of these patients never return to their baseline cognitive, physical, and psychological function. With more than 10 million inpatient procedures occurring annually in the United States, the literature would suggest that there are nearly one million episodes of delirium – many of them potentially preventable^18,19^ – with the resultant downstream effects.^20^ An intervention that can mitigate these downstream effects would have a significant impact on the recovery of a large number of patients undergoing major surgery.

**Figure 1:**
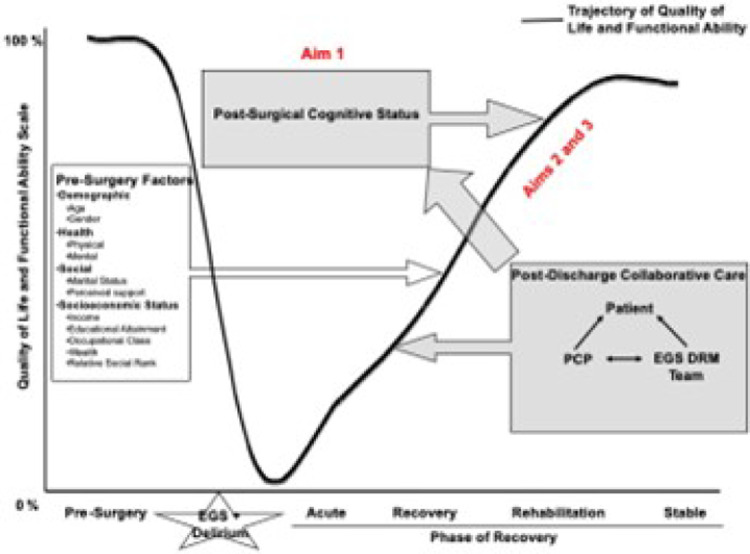
Conceptual model for Delirium and Neuropsychological Recovery among Emergency General Surgery Survivors (DANE) Recovery Model. Abbreviations: EGS, emergency general surgery; PCF; primary care provider; EGS DRM, emergency general surgery delirium recovery model.

**Figure 2:**
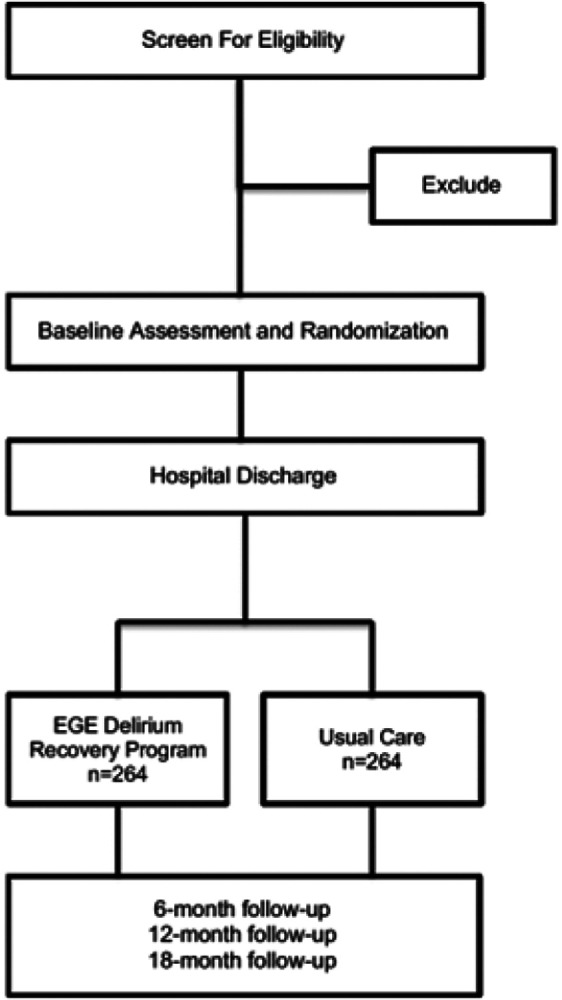
DANE Recovery Model Study Design.

Our conceptual model for this trial is based on that proposed by Wilson and Clearly,^10^ which focuses on factors that influence postsurgical quality of life and recovery trajectory. This model describes an abrupt decline in cognitive, physical, and psychological function in postoperative delirium survivors which is then followed by an upward trend during four phases of recovery: acute, recovery, rehabilitation, and stability. This intervention targets the all-too-common fragmentation of care that occurs in the first few months of surgery. Micro-measurements during this vulnerable period are a key component of a feedback process that identifies and intervenes early on those patients who exhibit signs of an altered recovery trajectory. We expect that the intervention will positively impact cognitive function and decrease downward pressure on the quality of life and functional recovery trajectory (Fig. 1).

A major strength of this study is in its innovative intervention which begins after the patient is discharged following surgery. This is an especially vulnerable time, particularly the first three months, following a major operation. Historically, collaborative care interventions have focused on populations which require chronic care management (e.g., dementia), and this study will determine the feasibility of doing this in a dynamic surgical recovery mode. Second, this intervention is primarily delivered via a secured telemedicine-based healthcare delivery platform. This increases access in settings where this limited, such as a pandemic setting and, especially in states like Wisconsin and Indiana, with patients that have limited ability to travel or a rural setting. Third, this trial highlights and acknowledges the need to consider surgical recovery beyond those outcomes that are typically tracked (e.g., short term inpatient morbidity). These are outcomes that are important to patients and their families, inform the surgical consent process, and can help in setting expectations for recovery. Interventions such as this one seek to address the problem of care fragmentation, which may lead to decreased resource utilization in addition to improving outcomes. Finally, as part of this trial and from our earlier and ongoing work with collaborative care interventions, we are continuing to refine and develop processes and tools that enable more reliable, long term follow up in older adults following surgery. We believe that these tools will benefit surgical patients on an ongoing basis after the study period has passed.

Limitations of this study include the setting which, though multi-center across both urban, suburban, and rural settings, is limited to the Midwest. This study does not account for any existing coordinated care plans that may be in place in our health systems.

### Trial Status

Recruitment and enrolling will begin 2/2023 with anticipated study end date of 1/2028. Protocol version 7.0 (6/2/2023). Study # NCT05373017 (clinicaltrials.gov).

## Figures and Tables

**Table 1.** Study Timeline

	Year 1				Year 2				Year 3				Year 4				Year 5			
	1	2	3	4	1	2	3	4	1	2	3	4	1	2	3	4	1	2	3	4
Start-up (hire/train staff, make study manuals, create data entry system)	X	X																		
Enrollment & randomization		X	X	X	X	X	X	X	X	X	X									
Deliver interventions			X	X	X	X	X	X	X	X	X	X	X	X	X					
Outcome measures			X	X	X	X	X	X	X	X	X	X	X	X	X	X	X	X	X	
Data entry & management			X	X	X	X	X	X	X	X	X	X	X	X	X	X	X	X	X	
Analyze data & submit report to DSMB				X	X	X	X	X	X	X	X	X	X	X	X	X	X	X	X	X
Present results at national meetings																		X	X	X

**Table 2.** Schedule of Activities

	Screening Day 14–0	Enrollment/ Baseline Day 0	Acute Stage Month 0 – 1	Recovery State Month 2– 6	6-Month Follow-up Week 21 – 31	Maintenance Stage Month 7 – 12	12- Mon up W 57
RA screens for eligibility and development of delirium	X						
Daily Delirium Assessment: RASS and CAMICU-7^a^	Xa						
DAST-10^a^	Xa						
Informed consent	X						
Demographics		X					
Behavior Checklist		Xb			X		X
RBANS		Xb			X		X
TMTs A & B		Xb			X		X
SCWT		Xb			X		X
IUTT		Xb			X		X
SPPB		Xb			X		X
SF-36		Xb			X		X
GAD-7		Xb			X		X
PHQ-9		Xb			X		X
Delirium Experience Questionnaire		Xb					
Lawton-Brody IADL		Xb					
Katz ADL		Xb					
QDRS		Xb					
Randomization^c^		Xc					
Medical history		X					
AE review and evaluation		X	X	X	X	X	X
Initial Case Review^d^			Xd				
First virtual visit with CC^d^			Xd				
Development of Recovery Care Plan^d^			Xd				
Second virtual visit with CC^d^			Xd				
Interaction Period^d^			Xd	Xd	Xd	Xd	

a: RASS, CAM-ICU-7 and DAST-10 performed as needed during screening to confirm eligibility. RA may perform CAM-ICU-7 up to twice daily.

b: RA will obtain baseline measurements *ideally* within 48 hours of anticipated hospital discharge *or* up to four weeks after discharge.

c: Randomization to occur *after* screening, consent and completion of baseline assessments, *ideally* within 48 hours of anticipated hospital discharge *or* up t

d: EGS Delirium Recovery Model only
